# Risk factors associated with colistin resistance in carbapenemase-producing *Enterobacterales*: a multicenter study from a low-income country

**DOI:** 10.1186/s12941-023-00609-8

**Published:** 2023-08-02

**Authors:** Soria-Segarra Claudia, Soria-Segarra Carmen, Diaz Andrés, Miranda-Ayala Marcela, Cevallos-Apolo Kerly, Bombón Moreno Bryan, Chuzan J. John, Gutierrez-Fernández José

**Affiliations:** 1 Sosecali. Medical Services, Guayaquil, EC 090308 Ecuador; 2 Faculty of Medical Sciences, Guayaquil University, Guayaquil, Ecuador; 3 Department of Microbiology, School of Medicine and PhD Program in Clinical Medicine and Public Health, University of Granada & ibs, Granada, Spain; 4 Department of Internal Medicine, School of Medicine, Universidad Católica de Santiago de Guayaquil, Guayaquil, Ecuador; 5 Hospital Guayaquil, Guayaquil, Ecuador; 6 Omni Hospital, Guayaquil, Ecuador; 7 Hospital de Infectología Dr. José Daniel Rodríguez Maridueña, Guayaquil, Ecuador; 8 Present Address: Department of Microbiology, Hospital Virgen de las Nieves, Institute for Biosanitary Research-Ibs, Granada, Spain; 9 Department of Microbiology, Hospital Alcívar, Guayaquil, Ecuador; 10 Department of Human Biology, University of Toronto, Toronto, Canada

**Keywords:** Colistin, Resistance, Carbapenemase-producing, *Enterobacterales*, Risk factors

## Abstract

**Purpose:**

The aim of this study was to assess the risk factors for colistin-resistant carbapenemase-producing *Enterobacterales* (CR-CPE), and describe the mortality associated with this organism, in a low-income country.

**Methods:**

A descriptive, observational, and prospective multicenter study was carried out in Guayaquil, Ecuador. All patients with carbapenem-resistant *Enterobacterales* admitted between December 2021 and May 2022 were enrolled. Infection definitions were established according to the Centers for Disease Control and Prevention (CDC) protocols. The presence of carbapenemase-producing *Enterobacterales* was confirmed with a multiplex PCR for *bla*_KPC,_ *bla*_NDM,_ *bla*_OXA-48,_ *bla*_VIM,_ and *bla*_IMP_ genes. MCR-1 production was studied molecularly, and MLST assays were carried out.

**Results:**

Out of 114 patients enrolled in the study, 32 (28.07%) had at least one positive sample for CR-CPE. *Klebsiella pneumoniae* ST512-KPC-3 was the most frequent microorganism isolated. Parenteral feeding, β-lactamase inhibitor use, recent hemodialysis, and renal failure were all considered independent risk factors for carrying CR-CPE. A mortality of 41.22% was detected, but we could not find any difference between colistin-resistant and colistin-susceptible CPE. MCR-1 production was not detected in any of the isolates studied.

**Conclusion:**

A significant burden for CR-CPE was found in a South American country that was mainly caused by the high-risk clone *K. pneumoniae* ST512-KPC-3 and not mediated by mcr-1 production. Its acquisition involved parenteral feeding, β-lactamase inhibitor use, recent hemodialysis, and renal failure as independent risk factors, demonstrating the critical need for infection prevention and stewardship programs to avoid dissemination to other countries in the region.

**Supplementary Information:**

The online version contains supplementary material available at 10.1186/s12941-023-00609-8.

## Introduction

The escalating proportion of healthcare-associated infections (HAI) caused by multidrug-resistant organisms (MDROs) constitutes a major health threat worldwide. Its economic burden is estimated at USD 1.1 billion per year, including more than 400,000 inpatient days and more than 10,000 deaths [1]. One of the leading causes of MDROs (multidrug-resistant organisms) infections are carbapenemase-producing *Enterobacterales*, microorganisms that have shown a rapid increase in prevalence during the last few years in developing countries [2–4].

The limited antimicrobial treatment options and insufficient development of new antibiotics against these microorganisms have forced physicians to use “last resort” drugs such as colistin [5]. As a consequence of this increase use of colistin, there has been a rise in the number of reports of colistin-resistant *Enterobacterales* [4, 6–9]. Colistin resistance is based on two principal mechanisms: the most frequent is a chromosomally encoded mechanism, whereas a less common form is through plasmid-mediated colistin resistance genes, such as *mcr* [10]. Although clinical experience with polymyxins began 40 years ago and therapeutic use for MDROs has dramatically increased in recent years, sparse data exist on the baseline prevalence and risk factors for colistin resistance among *Enterobacterales*; data from developing countries is particularly lacking [4, 8, 11].

In Ecuador, Ortega-Paredes et al. reported the first clinical isolate of colistin-resistant *Escherichia coli* harboring *mcr*-1 in 2016 [12]. Since then, *mcr*-1 in *E. coli* and *Klebsiella pneumoniae* has been described in Ecuador in animals from rural farms [13]. Therefore, this study aimed to assess the epidemiology and risk factors associated with colistin-resistant carbapenemase-producing *Enterobacterales* (CR-CPE) and describe the mortality in a low-income country.

## Methodology

A descriptive, observational, and prospective multicenter study was carried out in six private and public hospitals in Guayaquil, Ecuador. All patients admitted with carbapenem-resistant *Enterobacterales* between December 2021 and May 2022 were enrolled. Patient information was collected from electronic medical records, and infection definitions were established according to the Centers for Disease Control and Prevention (CDC) protocols [14].

The cases were patients infected or colonized with CR-CPE. All asymptomatic carriers were considered as colonized. The control group consisted of patients who tested negative for CR-CPE. The variables studied were: (1) sociodemographic data, (2) comorbidities, (3) Charlson’s severity index, (4) immunosuppression, (5) presence of invasive devices, (6) exposure to antimicrobials in the 90 days prior to CR-CPE screening, and (7) hospitalization unit.

### Microbiological characterization

All carbapenem-resistant *Enterobacterales* isolates were identified by the relevant microbiology unit and were collected and processed according to the protocols established by each institution. Carbapenem resistance was defined as an isolate categorized as intermediate or resistant to meropenem, imipenem, or ertapenem, according to Clinical Laboratory Standards Institute (CLSI) breakpoints.

Strains included for further study were stored at each hospital unit in Stuart Medium at room temperature until processing in a reference laboratory. Only one clinical isolate from each patient was studied, with preference given to those from sterile sites or with the highest resistance phenotype observed.

All isolates received at the reference laboratory were cultured in MacConkey agar (Becton–Dickinson, England) (16–18 h; 35 °C) to check their viability and purity. Bacterial identification was made using the Vitek 2 System (GN-Card) (BioMérieux, France) and conventional biochemical tests.

Carbapenem resistance was screened with CHROMagar Super Carba (CHROMagar, France) and confirmed with the disk diffusion test for meropenem and ertapenem according to CLSI guidelines. Further characterization of carbapenemase production was undertaken with the modified inactivation carbapenem method, following the methodology previously described [15, 16].

### Antimicrobial susceptibility profile

The minimal inhibitory concentration (MIC) of colistin was determined using broth microdilution (CBM) as described in the CLSI document M07-A9 [15]. Analytical-grade colistin sulphate (Sigma-Aldrich Code C2700000, batch 3.0) and Mueller Hinton broth with cation adjustment (Thermo-Fischer Scientific, United Kingdom) were used. The concentration range was 0.5–8 µg/mL, and CLSI breakpoints were used to define colistin resistance (MIC values ≥ 4 µg/mL) [16].

Susceptibility tests for aztreonam, ceftriaxone, cefepime, piperacillin/tazobactam, ciprofloxacin, amikacin, gentamicin, trimethoprim/sulfamethoxazole, colistin, tigecycline, ceftazidime/avibactam, and meropenem/vaborbactam were performed using disk diffusion. The results were interpreted using the CLSI breakpoints [16]. The U.S. Food and Drug Administration (FDA) breakpoints were used for tigecycline (https://www.fda.gov/drugs/development-resources/tigecycline-injection-products): for Fosfomycin IV, the EUCAST breakpoints were used (https://www.eucast.org/clinical_breakpoints). The intermediate category was interpreted as resistant to susceptibility profile analysis.

*E. coli* ATCC 25922, *Pseudomonas aeruginosa* ATCC 27853, and *K. pneumoniae* BAA ATCC 1705 were used for quality control of the bacterial identification tests and chromogenic agar and susceptibility tests. *P. aeruginosa* ATCC 27853 and *E. coli* AR Bank #0349 were used for the quality control of the CBM.

### Molecular carbapenemase identification and mcr-1 detection

A multiplex polymerase chain reaction (PCR) to detect the presence of *bla*_KPC,,_ *bla*_NDM,_ *bla*_OXA-48,_ *bla*_VIM,_ and *bla*_IMP_ was used to confirm the carbapenemase type in all CPE cases [17]. The *mcr*-1 gene was studied using a previously described multiplex PCR [18].

### Clonality study

Clonal relatedness was studied in colistin-resistant *bla*_KPC_-positive *Klebsiella pneumoniae* using ERIC-PCR, following the methodology previously described [3]. Isolates with 70% similarity in their electrophoretic pattern were not further analyzed.

### Multilocus sequence typing (MLST) and determination of the KPC variant

KPC-positive colistin resistant *K.pneumoniae* with different electrophoretic patterns were studied with MLST using the protocol described at https://bigsdb.pasteur.fr/klebsiella/primers-used/ and the sequence type assigned with the Klebsiella Pasteur MLST database (https://bigsdb.pasteur.fr/cgi-bin/bigsdb/bigsdb.pl?db=pubmlst_klebsiella_isolates). Additionally, for KPC variant identification, the primers UNIKPCF and UNIKPCR were used [18]. Twenty-one strains of *Klebsiella pneumoniae* were sequenced at Macrogen INC., Korea. The quality of the chromatograms of each sequence was analyzed using the bioinformatic tools FinchTV and SnapGene. Alignment of sequences was performed with MEGA 11 software. Variants were also analyzed with the basic local alignment search tool (BLAST) database (https://blast.ncbi.nlm.nih.gov/Blast.cgi?PROGRAM=blastn&PAGETYPE=BlastSearch&LINKLOC=blasthome). Sequence type (ST) numbers were determined using the public MLST website at (http://pubmlst.org/kpneumoniae).

### Statistical analysis

The analyses were carried out with the statistical package IBM SPSS version 28. Descriptive statistics were used, representing the absolute and relative variables of the qualitative variables as well as measures of central tendency and variability for the quantitative variables.

In terms of inferential statistics, a bivariate analysis was performed to determine the variables to be considered in the multivariate analysis. The Mann–Whitney test was used for quantitative variables (verifying non-normality), whereas the chi-square and Fisher Tests were applied to qualitative variables.

Multivariate logistic regression analysis was used to predict colistin resistance. Statistical significance of the comparisons of proportions, means, and predictor variables was established as *p* < 0.05.

### Ethical statement

This study was approved by the “Comite de Etica del Hospital Clinica Kennedy “[HCK-CEISH-19-0016]. Informed consent was waived due to the study design.

## Results

### CR-CPE clinical data

Out of 114 patients enrolled in the study, 32 (28.07%) had at least one positive sample for CR-CPE. Eleven patients (34.38%) were colonized, and 21 (65.62%) were infected with CR-CPE. A urinary tract infection was the most frequent disease, accounting for 38.10% (n = 8), followed by ventilator-associated pneumoniae (33.33%, n = 7), catheter-related bloodstream infections (9.52%, n = 2), surgical site infections (4.76%, n = 1), and others (4.92%, n = 3). The median time from admission to CR-CPE detection was 32 days. Table 1 shows the patient clinical characteristics; the diseases included in the immunosuppression variable did not show any statistically significant difference between patients with CR-CPE and colistin-susceptible carbapenemase-producing *Enterobacterales* (CS-CPE) (human immunodeficiency virus (0 (0.0%) vs. 6 (7.32%); p = 0.19), neutropenia (1 (3.23%) vs. 5 (6.1%); p = 1.00), steroid use (0 (0%) vs. 2 (2.44%); p = 1.00), chemotherapy/radiotherapy (2 (6.45%) vs. 6 (7.32%); p = 1.00).

**Table 1:** Clinical characteristics of hospitalized patients

Clinical characteristics	Total n = 114	Colistin-resistance		p-value
		Yes n = 32	No n = 82	
Age [median (IQR)]	56 (29–72)	61 (28–73)	50 (29–71)	0.297
Sex [n (%)]				
Female	40 (35.09)	8 (25)	32 (39.02)	0.159
Male	74 (64.91)	24 (75)	50 (60.98)	
Charlson [median (IQR)]	2 (0–4)	2 (1–6)	2 (0–3)	0.143
APACHE [median (IQR)]	15 (9–21)	16 (13–22)	15 (6–21)	0.201
Diagnostic [n (%)]				
Neurological disease	35 (30.97)	11 (35.48)	24 (29.27)	0.524
Acute respiratory failure	24 (21.05)	6 (18.75)	18 (21.95)	0.706
Chronic heart failure	14 (12.28)	5 (15.63)	9 (10.98)	0.532
Renal failure	17 (14.91)	9 (28.13)	8 (9.76)	0.020^a^
Diabetes mellitus	13 (11.4)	5 (15.63)	8 (9.76)	0.512
Immunosuppresion	13 (11.61)	3 (9.68)	10 (12.35)	1.000
Gastrointestinal disease	11 (9,65)	2 (6.25)	9 (10.98)	0.725
Malignancy	8 (7.02)	3 (9.38)	5 (6.1)	0.684
Polytrauma	7 (6.14)	3 (9.38)	4 (4.88)	0.399
Sepsis	6 (5.26)	1 (3.13)	5 (6.1)	1.000
Comorbidities [n (%)]				
Renal failure	28 (24.78)	12 (38.71)	16 (19.51)	0.035^a^
Chronic heart failure	51 (45.13)	17 (54.84)	34 (41.46)	0.202
Diabetes mellitus	32 (28.57)	11 (35.48)	21 (25.93)	0.316
Malignancy	17 (15.04)	6 (19.35)	11 (13.41)	0.556
Neurological disease	16 (14.16)	2 (6.45)	14 (17.07)	0.227
Immunosuppresion	13 (11.5)	2 (6.45)	11 (13.41)	0.509
Chronic pulmonar disease	4 (3.6)	1 (3.23)	3 (3.75)	1.000
Length of hospital stay [median (IQR)]	39 (20–156)	44 (21–124)	38 (19–158)	0.709
Mortality [n (%)]	47 (41.23)	14 (43.75)	33 (40.24)	0.733

Guayaquil-Ecuador; ^a^ Statistical significance p > 0.05

*IQR* interquartile range

We did not find any difference between CR-CPE and CS-CPE according to the type of unit where the microorganisms were isolated. These units included: intensive care unit (14 (43.5%) vs. 42 (51.85%)), medical wards (10 (31.25%) vs. 20 (24.69%)), surgical wards (2 (6.25%) vs. 6 (7.41%)), emergency department (1 (3.13%) vs. 3 (3.7%)), neonatal ward (0 (0%) vs. (1.23%)), neonatal intensive care unit (1 (3.313%) vs. (7.41%)), pediatric intensive care unit (0 (0) vs. 1 (1.3%)), post-critical care (4 (12.5%) vs. 2 (2.47%); p = 0.48).

### Univariate analysis

Renal failure diagnosis and comorbidity, hemodialysis, previous infection with carbapenem-resistant *Acinetobacter baumannii*, total parenteral nutrition, and β-lactamase inhibitor (ampicillin/sulbactam, piperacillin/tazobactam) use were considered risk factors for CR-CPE in the univariate analysis (Tables 1 and 2).

**Table 2:** Univariate analysis risk factor for colistin-resistance carbapenemase-producing *Enterobacterales*

Variables	Total	Colistin resistance		p-value
		Yes	No	
		n = 32	n = 82	
	n = 114			
	n (%)	n (%)	n (%)	
Invase procedures				
Mechanical ventilator	74 (64.91)	23 (71.88)	51 (62.2)	0.331
Central venous catheter	76 (67.26)	21 (67.74)	55 (67.07)	0.946
Urinary catheter	90 (79.65)	25 (78.13)	65 (80.25)	0.801
Gastrostomy	16 (14.55)	5 (16.67)	11 (13.75)	0.763
Tracheostomy	46 (40.35)	16 (50)	30 (36.59)	0.19
Nasogastric tube	74 (64.91)	18 (56.25)	56 (68.29)	0.226
Hemodyalisis catheter	25 (22.12)	13 (41.94)	12 (14.63)	0.002^a^
Surgery	44 (38.94)	12 (38.71)	32 (39.02)	0.976
Total parenteral Nutrition	24 (21.43)	2 (6.67)	22 (26.83)	0.021^a^
Peripherical catheter	87 (77.68)	19 (63.33)	68 (82.93)	0.027^a^
Antimicrobials				
β-lactam-inhibitors	31 (27.19)	13 (40.63)	18 (21.95)	0.044^a^
Cephalosporins	32 (28.07)	8 (25)	24 (29.27)	0.649
Aztreonam	2 (1.75)	1 (3.13)	1 (1.22)	0.484
Carbapenem	56 (49.12)	16 (50)	40 (48.78)	0.907
Quinolone	27 (23.68)	7 (21.88)	20 (24.39)	0.777
Vancomycin	44 (38.6)	10 (31.25)	34 (41.46)	0.314
Clindamycin	17 (15.18)	5 (16.13)	12 (14.81)	1.000
Doxycycline	6 (5.26)	1 (3.13)	5 (6.1)	1.000
Tigecycline	11 (9.65)	4 (12.5)	7 (8.54)	0.499
Colistin	15 (13.16)	6 (18.75)	9 (10.98)	0.355
Aminoglycosides	20 (17.54)	5 (15.63)	15 (18.29)	0.736
Trimethoprim/sulfamethoxazole	9 (7.89)	2 (6.25)	7 (8.54)	1.000
Metronidazol	13 (11.4)	3 (9.38)	10 (12.2)	1.000
Macrolides	15 (13.51)	5 (15.63)	10 (12.66)	0.761
Linezolid	10 (9.01)	4 (12.5)	6 (7.59)	0.47
Microorganism isolated previous CR-CPE				
ESBL* E. coli*	5 (4.39)	0 (0)	5 (6.1)	0.32
ESBL *K. pneumoniae*	2 (1.75)	0 (0)	2 (2.44)	1.00
Carbapenem-resistant *A. baumannii*	5 (4.39)	4 (12.5)	1 (1.22)	0.021^a^
Methicillin-resistant *S. aureus*	7 (6.14)	3 (9.38)	4 (4.88)	0.399
*E. coli*	3 (2.63)	1 (3.13)	2 (2.44)	1.00
*K. pneumoniae*	2 (1.75)	0 (0)	2 (2.44)	1.00
*A. baumannii*	4 (3.51)	0 (0)	4 (4.88)	0.20
Methicillin- susceptible *S. aureus*	1 (0.88)	0 (0)	1 (1.22)	1.00
*P. aeruginosa*	1 (0.88)	0 (0)	1 (1.22)	1.00
*E. cloacae*	2 (1.75)	0 (0)	2 (2.44)	1.00
Otros	28 (24.56)	7 (21.88)	21 (25.61)	0.677
Previous exposures within 3 months				
Hospitalization	47 (41.96)	14 (43.5)	33 (41.25)	0.809
Inmunosupression	21 (18.58)	8 (25.0)	13 (16.05)	0.270
Intubation	17 (15.18)	4 (12.5)	13 (16.25)	0.774
Invasive procedures	21 (18.58)	4 (12.5)	17 (20.99)	0.296
Hemodialysis	9 (8.04)	6 (18.75)	3 (3.75)	0.016^a^

Chi-squared test or Fisher´s exact test

^a^Statistical significance p > 0.05

*CR-CPE* Colistin-resistant carbapenemase producing *Enterobacterales*, *ESBL* extended-spectrum beta-lactamase

### Multivariate analysis

Our multivariate analysis showed that renal failure diagnosis, hemodialysis, isolation of CR-CPE in the preceding 3 months, total parenteral nutrition, and β-lactamase inhibitor use were independent risk factors for CR-CPE (Table 3).

**Table 3:** Multivariate and Univariate analyses of risk factors associated with the development of colistin-resistant carbapenemase-producing *Enterobacterales* strains

Risk factors	Univariate		Multivariate	
	OR (IC95%)	p-value	OR (IC95%)	p-value
Renal failure diagnosis				
Renal failure	3.62 (1.25–10.46)	0.018^a^	0,.1 (0.11–2.48)	0.03^a^
Previous exposure within 3 months				
Hemodialysis	5.92 (1.38–25.39)	0.017^a^	3.45 (0.51–23.9)	0.03^a^
Invasive procedures				
Hemodialysis catheter	4.1 (1.65–10.79)	0.003^a^	3.20 (0.77–13.37)	0.110
Parenteral nutrition	0.20 (0.043–0.89)	0.034^a^	0.15 (0.02–0.94)	0.043^a^
Peripherical catheter	0.36 (0.4–0.91)	0.031^a^	0.39 (0.13–1.14)	0.084
Antimicrobial use				
Previous exposure to β-lactamase-inhibitor	2.43 (1.01–5.85)	0.047^a^	2.97 (1.07–8.24)	0.036^a^

Guayaquil-Ecuador

^*^p < 0.05

### Carbapenemase production characterization and colistin resistance

A total of 114 carbapenem resistant isolates were obtained. All were characterized as carbapenemase producers*. K. pneumoniae* was the most prevalent microorganism isolated (93.50%, n = 72), followed by *E. cloacae* (n = 3) and *K. aerogenes* (n = 2).*bla*_KPC_ was the most prevalent gene found in CPE; however, one *K. pneumoniae* isolate produced NDM. Thirty-two *K. pneumoniae* isolates were categorized as colistin resistant, and all were KPC producers. Detailed information on susceptibility profiles is given in Table 4.

**Table 4:** Susceptibility profile of colistin-resistant and colistin-susceptible carbapenemase producing-*Enterobacterales*

Antibiotic	*K. pneumoniae*			*E. aerogenes*			*E. cloacae*		
	Total	Colistin-resistance		Total	Colistin-resistance		Total	Colistin-resistance	
		Yes	No		Yes	No		Yes	No
	n = 84	n = 25	n = 54		n = 1	n = 2		n = 1	n = 2
				n = 3			n = 3		
	n (%)	n (%)	n (%)	n (%)	n (%)	n (%)	n (%)	n (%)	n (%)
Meropenem	0/84 (0)	0/25 (0)	0/54 (0)	0/3 (0)	0/1 (0)	0/2 (0)	0/3 (0)	0/1 (0)	0/2 (0)
Ertapenem	0/84 (0)	0/25 (0)	0/25 (0)	0/3 (0)	0/1 (0)	0/2 (0)	0/3 (0)	0/1 (0)	0/2 (0)
Aztreonam	0/84 (0)	0/25 (0)	0/25 (0)	0/3 (0)	0/1 (0)	0/2 (0)	0/3 (0)	0/1 (0)	0/2 (0)
Piperacillin/tazobactam	0/84 (0)	0/25 (0)	0/25 (0)	0/3 (0)	0/1 (0)	0/2 (0)	0/3 (0)	0/1 (0)	0/2 (0)
Amikacin	29/79 (36.7)	1/25 (4)	28/54 (52)	3/3 (100)	1/1 (100)	2/2 (100)	2/2 (100)	1/1 (100)	2/2 (100)
Gentamicin	48/79 ((60.75)	21/25 (84)	27/54 (50)	2/3 (66.67)	1/1 (100)	1/2 (50)	1/2 (50)	1/1 (100)	1/2 (50)
Ciprofloxacin	8/78 (10.25)	0/25 (0)	8/54(15)	1/3 (33.3)	0/1 (0)	1/2 (50)	1/2 (50)	0/1 (0)	1/2 (50)
Tigecycline	31/53 (58.49)	25/25 (100)	26/37 (70)	1/1 (100)	1/1 (100)	1/1 (100)	2/2 (100)	1/1 (100)	2/2 (100)
Trimethoprim-sulfamethoxazole	2/33 (6.06)	1/25 (4)	1/8 (13)	0/1 (0)	0/1 (0)		0/1 (0)	0/1 (0)	
Ceftazidime-avibactam	25/25 (100)	25/25 (100)		0/1 (100)	0/1 (100)		1/1 (100)	1/1 (100)	
Meropenem/vaborbactam	23/25 (92)	23/25 (92)					1/1 (100)	1/1 (100)	
Fosfomycin iv	5/25 (20)	5/25 (20)		0/1 (100)	0/1 (9)		0/1 (0)	0/1 (0)	

Values of minimal inhibitory concentration to colistin are detailed in Additional file 2: Table S1. Colistin MIC distribution.

### Molecular characterization of CR-CPE

Eleven electrophoretic patterns were found in colistin-resistant *Klebsiella pneumoniae* isolates (Additional file 1: Fig S1. Dendogram of ERIC PCR results). These were sequenced and analyzed to determine the KPC variant and the ST. ST512, which carried the KPC-3 variant, was predominant (90.90%, n = 10). Only one isolate of ST111 with the KPC-2 variant was detected. None of the other carbapenemase genes studied were positive, nor was the *mcr-1* gene.

## Discussion

Our 6 month surveillance confirmed a high prevalence of CR-CPE in Guayaquil, the most populated city of Ecuador. Reports from Brazil and other South American countries, including one from PAHO (the Pan-American Health Organization), have shown CR-CPE prevalence rates ranging from 4 to 38.5% [4, 8, 11, 19, 20], with *K. pneumoniae* as the most frequent microorganism isolated [21]. Since resistance to carbapenem in different institutions in Ecuador is elevated, colistin has become the first-line drug to treat infections caused by Gram-negative pathogens, particularly in the ICU [3]. This explains the increase in colistin use and may have contributed to the increasing colistin resistance rates.

High mortality rates occur in patients infected with CR-CPE and range from 9 to 38% [8, 20, 22–25]. However, we did not find statistical differences in mortality for CR-CPE vs. CS-CPE (p = 0.73). However, other published reports from Italy and Brazil did show a statistical difference in mortality rates in patients infected with CR-CPE vs. CS-CPE [22, 23, 25]. We hypothesized that this difference could be due to the presence of bloodstream infections in the Italian groups and the inclusion of neonates by the Brazilian team. Bloodstream infections produce higher mortality rates than urinary tract infections [22, 25–27], which was the most frequent infection identified in our data.

Our multivariate analysis showed renal failure, hemodialysis, parenteral nutrition, and β-lactamase inhibitor use as independent risk factors associated with CR-CPE. Other studies have described different comorbidities associated with CR-CPE, including neurological disease [28], chronic kidney disease [23], and Charlson score > 3 [22, 23, 28]. A multi-variate analysis revealed that renal failure was an independent predictor for CR-CPE in critically ill patients (OR 11.37, 95% IC CI 1.0–128.63) [23]. A relevant difference between studies could be that they were undertaken with different populations, healthcare facility levels, sample sizes, and methodologies.

Total parenteral nutrition has not been previously recognized as an independent risk factor for CR-CPRE, since most studies do not include this variable [5, 23, 28]. Nutrition support impacts the gastrointestinal microbiota, predisposing a trend towards greater abundance for pathogenic Proteobacteria (including the families of *Enterobacteriaceae, Pseudomonas,* and *Klebsiella*), which differs from healthy individuals who have high abundance of Bacteroidetes and Firmicutes [29].

Interestingly, isolation of carbapenem-resistant *Acinetobacter baumannii* was determined in the univariate analysis as a risk factor for CR-CPE. However, we could not find this association in the multivariate analysis, probably due to the small sample size. This suggests that controlling carbapenem use may help to mitigate the emergence of CR-CPE [30].

Prior antibiotic exposure is considered an important risk factor for colistin resistance [20, 21, 28, 31]. In our study, β-lactamase inhibitors were found to be independent predictors of CR-CPE. In our country, these antimicrobials are frequently use in patients with community onset infections, which could predispose multidrug-resistant microorganism isolation [28].

We found no link between colistin-resistant isolates and previous exposure to colistin [21, 22, 27, 28, 32]. However, the findings on this topic are still controversial [31, 33]; one study found a protective effect for the use of colistin–tigecycline combination therapy [34], whereas another showed a protective effect for aminoglycosides in acquired colistin-resistant strains [23]. In our institutions, colistin is used when a CPE infection is suspected and confirmed. Thus, previous colistin used was not frequent in our data.

The epidemiology of carbapenemase is dynamic [3, 35], and this is the first time that KPC-3 ST512 has been reported in our country. Despite having been previously reported, no OXA carbapenemase types were found in this study. The clinical implications of this are significant, since this knowledge influences the selection of empiric antibiotics. The most prevalent STs in the clonal group CG258 are ST512 and ST258. Notably, the emergence of the bla_*KPC*_-3 gene has been related to the spread of ST512 in Italy [36], Algeria, Israel [37], and Spain [38], has been linked to the coexistence of several *K. pneumoniae* subpopulations, and is particularly associated with outbreaks.

Plasmid-mediated colistin resistance, especially related to the *mcr-1* gene, is increasing in frequency in Ecuador, Argentina, Brazil, and Colombia [7, 12, 13] and is the most relevant mechanism in colistin resistance; however, it has mainly been described in *E. coli* [13]. Our sample was composed mostly of *K. pneumoniae,* where this resistance mechanism is rarely involved. Our study did not find this mechanism of resistance in any isolations, suggesting another mechanism could be involved. A recent meta-analysis published by Yusof et al. evaluated the prevalence of mutations in colistin-resistance genes worldwide and showed that the mutation most frequently reported was in the *mgrB* gene, followed by the *prmB* and *phoQ* genes, which supported the idea that a genetic mutation on chromosomal genes may be involved in our resistant isolates [10]. In South America and Europe, different countries have found chromosomal mutations to be the most frequent mechanism causing colistin resistance [11, 22–24, 39].

This study has several limitations. First, our sample size was insufficient and the statistical analysis did not have the power to extrapolate the findings to the entire population. Second, we were unable to distinguish the colistin resistance genetic etiology, which was different from the *mcr-1* gene. Finally, the results could not be extrapolated to CR-CPE harboring the *mcr-1* gene because non-CR-CPE strains carrying the gene were found in our study. Despite these limitations, our research provides important data about the risk factors associated with CR-CPE in low-income developing nations and warns of their transferability to neighboring South American countries.

In conclusion, a significant burden for CR-CPE was found in a South American country; this was mainly caused by *K. pneumoniae* ST512-KPC-3 and parenteral feeding, β-lactamase inhibitor use, recent hemodialysis, and renal failure, which were all independent risk factors for carrying CR-CPE. This article demonstrated the critical need for infection prevention and stewardship programs and emphasizes the importance of researching other colistin-resistant mechanisms in underdeveloped nations, where most hospital laboratories do not regularly screen for colistin susceptibility.

## Supplementary Information

**Additional file 1: Figure S1. **Dendogram of ERIC PCR Results.

**Additional file 2: Table S1. **Colistin MIC distribution

## Data Availability

The datasets generated during and/or analyzed during the current study are available from the corresponding author on reasonable request.

## Abbreviations

CR-CPE: Colistin-resistant carbapenemase-producing *Enterobacterales*
CDC: Centers for disease control and prevention
HAI: Healthcare-associated infections
MDROs: Multidrug-resistant organisms
CLSI: Clinical Laboratory Standards Institute
MIC: Minimal inhibitory concentration
ATCC: American type culture collection
PCR: Polymerase chain reaction
CR: Colistin resistance
MLST: Multilocus sequence typing
BLST: Basic local alignment search tool
CS-CPE: Colistin-susceptible carbapenemase-producing *Enterobactales*
CPE: Carbapenemase-producing *Enterobacterales*
ST: Sequence type
