# Supplementary figures and images for: Risk factors associated with colistin resistance in carbapenemase-producing Enterobacterales: a multicenter study from a low-income country
Source: Ann Clin Microbiol Antimicrob. 2023 Aug 2;22:64. doi: 10.1186/s12941-023-00609-8 (PMC10398925; doi:10.1186/s12941-023-00609-8)

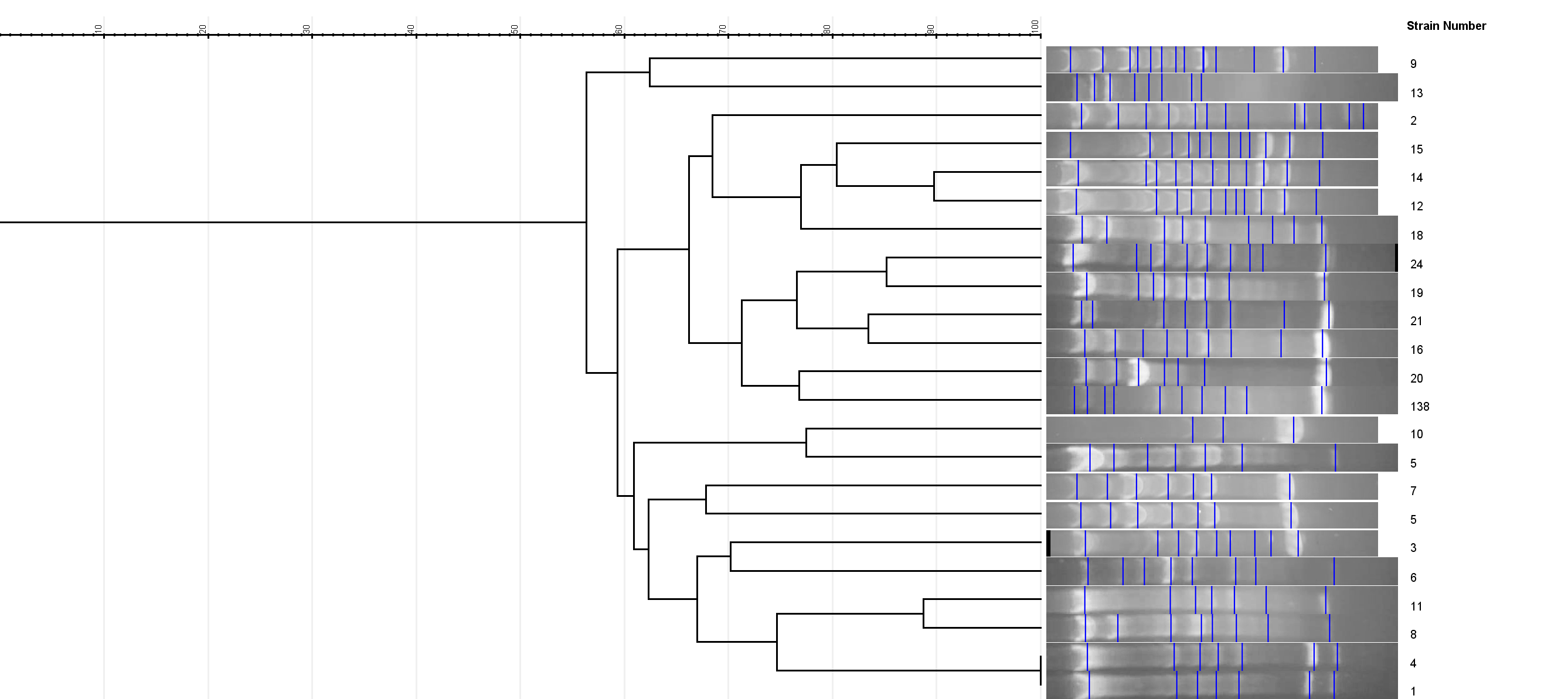

Supplement: Supplementary file 1 — Additional file 1: Figure S1. Dendogram of ERIC PCR Results. [file 12941_2023_609_MOESM1_ESM.tif]
