# Supplementary material for: Risk factors associated with colistin resistance in carbapenemase-producing Enterobacterales: a multicenter study from a low-income country
Source: Ann Clin Microbiol Antimicrob. 2023 Aug 2;22:64. doi: 10.1186/s12941-023-00609-8 (PMC10398925; doi:10.1186/s12941-023-00609-8)
Supplement: Supplementary file 2 — Additional file 2: Table S1. Colistin MIC distribution [file 12941_2023_609_MOESM2_ESM.docx]

|  | **MIC DISTRIBUTION  † (**  *µ*g**/mL)** | | | | | |  |
| --- | --- | --- | --- | --- | --- | --- | --- |
| **Microorganism** | **< 0.5** | **0.5** | **1** | **2** | **4** | **> = 8** | **Total** |
| ***K. aerogenes*** | 3 |  |  |  |  | 1 | 4 |
| ***E.cloacae*** |  |  | 1 | 1 | 1 |  | 3 |
| ***E. coli*** | 3 |  |  |  |  |  | 3 |
| ***K.pneumoniae*** | 51 | 3 | 8 | 12 | 24 | 6 | 104 |
|  | †Results obtained by broth microdilution method | | | | |  |  |
|  | Black line detailed breakpoint according to CLSI | | | | |  |  |

**Table S1.** Colistin MIC distribution
